# Assessment of the First Commercial ELISA Kit for the Diagnosis of* Theileria annulata*

**DOI:** 10.1155/2015/787812

**Published:** 2015-11-10

**Authors:** Amira A. T. Al-Hosary, Jabbar Ahmed, Ann Nordengrahn, Malik Merza

**Affiliations:** ^1^Department of Animal Medicine (Infectious Diseases), Faculty of Veterinary Medicine, Assiut University, Assiut 71526, Egypt; ^2^Institute of Parasitology and Tropical Veterinary Medicine, Faculty of Veterinary Medicine, Free University of Berlin, 14195 Berlin, Germany; ^3^Boehringer Ingelheim Svanova, Uppsala Business Park, P.O. Box 1545, 75145 Uppsala, Sweden

## Abstract

The present study assesses the efficacy of SVANOVIR* Theileria annulata*-Ab, the first commercial ELISA kit for the diagnosis of* Theileria annulata* infection in cattle based on a recombinant protein known as* T. annulata* surface protein (TaSp). As a reference test, a polymerase chain reaction (PCR) assay depending on* T. annulata* merozoite surface antigen (Tams-1) was applied. A total of 468 blood samples as well as serum samples were randomly collected from cattle and tested in the PCR as well as in the ELISA developed in this study. Moreover, all samples were also analyzed by conventional Giemsa-stained blood smear. The results of this study revealed a good correlation between the results obtained by PCR and the ELISA, whereas all PCR positive samples scored correctly positive in the ELISA and 73 of the 125 PCR negative samples scored correctly negative. Taken together, a sensitivity of 91.25% and a specificity of 78.4% were recorded, when compared to the PCR data. In conclusion, the SVANOVIR* Theileria annulata*-Ab is a suitable diagnostic assay for use in the diagnosis and epidemiological surveys of* Theileria annulata* infection in chronic and carrier animals.

## 1. Introduction

Tropical theileriosis is a tick-borne disease caused by the protozoan parasite* Theileria annulata* and is transmitted by the tick vector* Hyalomma anatolicum* [1, 2]. This disease is a major constraint on livestock production in areas at risk of the infection. Early and accurate diagnosis of the disease is crucial for the control of the infection. During the acute phase of the infection, when the level of parasitemia is high, it is easier to diagnose the infection by microscopical examination of Giemsa-stained thin blood smear, which is the most common traditional method for diagnosis of blood parasites. However, serological assays are more suitable for the diagnosis of the disease during the chronic phase of the infection, where the animals serve as carriers. These animals have higher antibody titers, while the level of parasitemia is low and microscopically hardly undetectable [3]. Molecular identification tools are accordingly the best techniques [4–6]. The present study aims at evaluating the reliability of the commercial ELISA kit depending on its sensitivity and specificity in the diagnosis of* Theileria annulata* infection in naturally infected cattle. This validation was performed in comparison with the Tams-1 PCR.

## 2. Materials and Methods

### 2.1. Study Areas and Samples Collection

The present study was conducted on 468 cattle samples which were collected from different localities in Al-Wadi Al-Jadid governorate, Egypt. Two whole blood samples were collected from the jugular vein using vacutainer tubes, one without anticoagulant for serum preparation and the other one containing ethylene diamine tetra acetic acid (EDTA) as anticoagulant for DNA extraction. Blood smears were prepared immediately from blood obtained by ear vein puncture.

### 2.2. Conventional Diagnosis Based on Giemsa-Stained Blood Smear

Three thin blood smears from each animal were examined for confirmation of the infection through detection of intraerythrocytic piroplasms.

### 2.3. Serological Diagnosis Using SVANOVIR* Theileria annulata*-Ab

Serological diagnosis was carried out using SVANOVIR* Theileria annulata*-Ab (Boehringer Ingelheim Svanova, Uppsala, Sweden; The kit is not yet released to the market) that utilizes a recombinant* Theileria annulata* surface protein (TaSP) antigen and as recommended by the manufacturer. Measurement of the optical density (OD) at 450 nm was carried out using Multiscan Spectrum (Thermo Electron Corporation, Finland) ELISA reader. Results were presented as percent positivity (PP) that were calculated as the mean OD values of duplicate tested samples divided by the mean OD values of the positive control and multiplied by 100. The cut-off was set at 46 PP with specificity and sensitivity of 100% by using MedCalc Software bvba, Belgium.

### 2.4. Molecular Diagnosis Based on Tams-1 Target Based PCR

#### 2.4.1. DNA Extraction

DNA extraction from whole blood was carried out according to the manufacturer's instructions of the commercial kits (QIA amp blood kit, Qiagen, Ltd, UK, Cat. No. 51104).

#### 2.4.2. Tams-1 Target-Based PCR

DNA amplification by using Tams-1 primer for the standard PCR, primer Tams-1 F (5∖ATG CTG CAA ATG AGG AT) and Tspms1 R (5∖GGA CTG ATG AGA AGA CGA TGA G), to amplify 785 bp fragment of the* Theileria annulata* 30 KDa major merozoite surface antigen gene [7]. The specific bands were detected by high performance ultraviolet trans-illuminator, (UV, INC, UK) and the image of the PCR products containing the DNA sequence of 785 bp was visualized using DOC-ItLS, image acquisition software (UVP, INC, UK).

## 3. Results and Discussion

**Figure 1:**
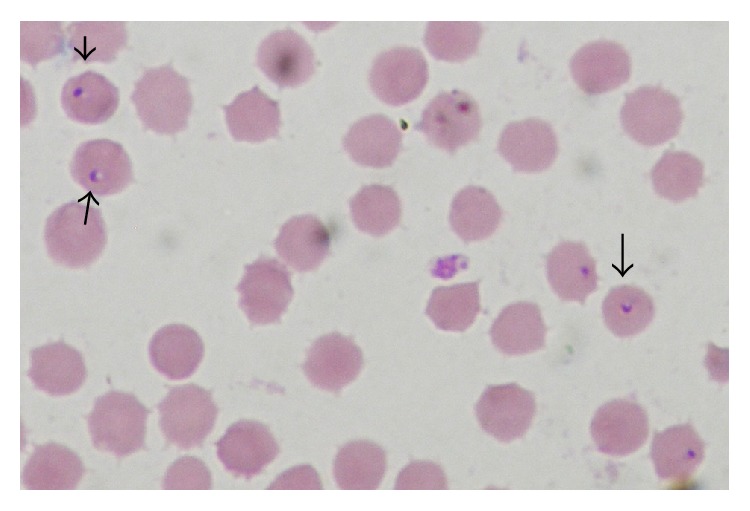
Blood smear stained with Giemsa stain demonstrating intracellular trophozoite “signet ring” of* Theileria annulata* (×100) (arrows).

**Figure 2:**
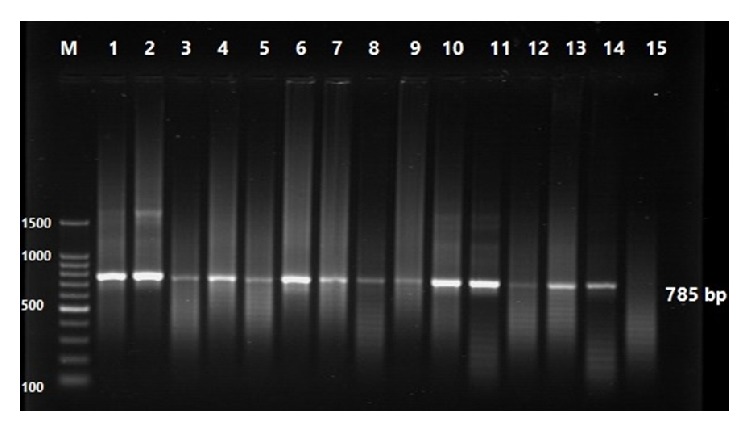
(Tams-1) target based PCR amplified DNA from* Theileria annulata* infected animals M, 100 bp DNA Marker Lanes 1 : 14 positive samples yielded PCR products at 785 bp, lanes 15 negative sample.

Molecular techniques are considered as the most sensitive and specific diagnostic assays for the diagnosis of* Theileria annulata* infection [4, 6, 8]. Accordingly, Tams-1 PCR was used as a reference test to evaluate the reliability of the SVANOVIR* Theileria annulata*-Ab under field conditions (Figure 2). For comparison, samples were also analyzed with Giemsa blood smear (Figure 1). Tabidi et al. [9] and Salih et al. [10] have previously reported poor sensitivity using conventional methods. Moreover, problems regarding cross-reaction between* Theileria* species were also recorded.

In order to avoid cross-reactions in the SVANOVIR* Theileria annulata*-Ab, the TaSp protein used as antigen in the present study was examined for possible cross-reactions with other pathogens. No cross-reactions were found using the TaSp recombinant protein in ELISA analyzing anti-*Babesia* sera or sera from animals experimentally infected with* Theileria mutans* and* Theileria parva*. [11, 12]. No cross-reactivity was recorded when Ta-LFD test was performed examining sera from animals experimentally infected with* Babesia bovis, Babesia bigemina, Trypanosoma brucei, Anaplasma marginale, Theileria mutans, and Theileria parva* as well as serum from an animal testing positive in an ELISA for detection of infection with* Theileria lestoquardi*. Interestingly, none of the serum samples of* Theileria annulata*-negative control animals could be positively tested [13]. Moreover, the SVANOVIR* Theileria annulata*-Ab was validated against monospecific sera to* Trypanosoma brucei, Anaplasma marginale, Babesia bovis, Babesia bigemina, and Theileria parva*. Antibodies to these pathogens were not detected in the ELISA (data not shown). The results of our study confirm a similar pattern where the Tams-1 PCR confirmed* Theileria annulata* infection in 343 out of 468 samples (73.29%). The SVANOVIR* Theileria annulata*-Ab scored 340 out of the 468 samples as positive (72.65%) while the Giemsa-stained blood smear only confirmed 145 positive cases (30.98%). The Tams-1 PCR revealed 125 samples as negative. In the SVANOVIR* Theileria annulata*-Ab 128 samples turned to be negative, while the Giemsa-stained blood smear scored as many as 323 negative samples (Table 1). In comparison 313 of the 343 Tams-1 PCR positive samples turned positive in the SVANOVIR* Theileria annulata*-Ab while 98 of the 125 PCR negative samples became ELISA negative. The SVANOVIR* Theileria annulata*-Ab scored 27 samples false positive and 30 samples false negative in comparison to the Tams-1 PCR giving a sensitivity of 91.25% and a specificity of 78.4%. The Giemsa blood smear, on the other hand, missed more than half of the Tams-1 PCR positive samples, giving a sensitivity of 42.27%, while all 125 PCR negative samples scored correctly negative (specificity 100%) (Table 3). Considering the fact that PCR detects very small amount of DNA, it will detect also a low level of parasitemia. Although the ELISA shows a lower specificity (78.4%) compared to the blood smear (100%), it shows a better correlation to the PCR. In the ELISA 57 samples obtained a different status while in the blood smear as many as 198 samples scored incorrectly in comparison to the PCR. The ELISA is therefore considered as an excellent tool in detecting* Theileria annulata* antibodies, especially in chronic and carrier animals. During the acute phase of the disease it is recommended to use another assay since the ELISA is unlikely to detect antibodies during the first week of infection. Our results confirmed that the thin blood smear is a specific but moderately sensitive diagnostic test (Table 2). Although it is a cheap, easy, and fast test, it is limited for detection of the acute infection, when the level of parasitemia is high enough to be detected microscopically. These findings are in agreement with previous results obtained by [10, 14]. Predictive values usually give an indication about the accuracy of a result in an assay. The Positive Predicted Value (PPV) scores the probability that the disease is present when the test is positive while the Negative Predictive Value (NPV) scores the probability that the disease is not present when obtaining a negative test result. SVANOVIR* Theileria annulata*-Ab revealed a relatively high PPV, (92%), and as expected did also the Giemsa blood smear, 100% (Table 3). These findings indicate that during further investigations the SVANOVIR* Theileria annulata*-Ab has the ability to correctly identify true positive cases as being positive. The NPV of SVANOVIR* Theileria annulata*-Ab was (76.56%) and is considered relatively high as compared to 38.70% for the thin blood smear. These results clearly indicate that the SVANOVIR* Theileria annulata*-Ab has a much higher ability to detect true negative cases compared to thin blood smear. The combined predictive values were 87.82% and 57.69% for the SVANOVIR* Theileria annulata*-Ab and the Giemsa-stained blood smear, respectively.

## 4. Conclusions

The SVANOVIR* Theileria annulata*-Ab is an excellent tool for detection of antibodies to the parasite in chronic and carrier animals. Microscopic examination of Giemsa-stained thin blood smear is helpful in day-to-day examination during acute infection, making quick decisions and confirming the infection. This important information is crucial for decision makers to start with a control program or not. The SVANOVIR* Theileria annulata*-Ab is highly recommended for epidemiological studies of tropical theileriosis in cattle, especially to detect chronic and carrier cases.

## Figures and Tables

**Table 1:** Confirmed *Theileria annulata *infection by the three diagnostic tools.

Diagnostic assay	Positive	%	Negative	%
Giemsa-stained blood smear	145	30.98	323	69.02
SVANOVIR *T. annulata*-Ab	340	72.65	128	27.35
Tams-1 PCR	343	73.29	125	26.71

**Table 2:** Results obtained using all three diagnostic techniques: conventional, serological, and molecular methods.

Results of both Giemsa-stained blood smear and SVANOVIR *T. annulata*-Ab	Tams-1 PCR+	Tams-1 PCR−	Total
Giemsa-stained blood smear+ SVANOVIR *T. annulata-*Ab+	115	0	115

Giemsa-stained blood smear+ SVANOVIR *T. annulata-*Ab−	30	0	30

Giemsa-stained blood smear− SVANOVIR *T. annulata-*Ab+	198	27	225

Giemsa-stained blood smear− SVANOVIR *T. annulata-*Ab−	0	98	98

Total	343	125	468

**Table 3:** Evaluation of conventional and SVANOVIR *T. annulata-*Ab for diagnosis of *Theileria annulata* in cattle. Cut-off 46 PP.

Diagnostic methods	Test results				Evaluation parameters (%)				
	TP^a^	TN^b^	FP^c^	FN^d^	Sensitivity	Specificity	PPV^e^	NPV^f^	CPV^g^
Giemsa-stained blood smear	145	125	0	198	42.27	100	100	38.70	57.69

SVANOVIR *T. annulata*-Ab	313	98	27	30	91.25	78.4	92	76.56	87.82

Tams-1 target based PCR were considered as the reference test.

^a^True positive, ^b^true negative, ^c^false positive, ^d^false negative, ^e^positive predictive value, ^f^negative predictive value, and ^g^combined predictive value.
